# Policy vs Business: Well-Designed Health-Related Food Policy Should Not Let Industry Marketing Undermine its Intended Effects: Comment on "Understanding Marketing Responses to a Tax on Sugary Drinks: A Qualitative Interview Study in the United Kingdom, 2019"

**DOI:** 10.34172/ijhpm.2023.7640

**Published:** 2023-02-26

**Authors:** Laura Cornelsen, Soledad Cuevas

**Affiliations:** Department of Public Health, Environments and Society, London School of Hygiene & Tropical Medicine, London, UK.

**Keywords:** Food industry, Marketing, Policy, Sugar, Tax

## Abstract

Health-related food policies, such as taxes on unhealthy food and drink, can act as catalysts for food industry to take action which may or may not align with the policy goal of improving population health. This commentary discusses the framework proposed by Forde et al in analysing the food industry marketing responses to the Soft Drink Industry Levy (SDIL), implemented in the United Kingdom in 2018. We suggest and discuss ways which may help broaden the use of the framework to other relevant policies. This includes widening the framework to cover strategies that may have not come up within the SDIL context but have been studied in other contexts. It would also be important to consider interactions between the strategies and with external factors influencing company decisions. Going forward, comprehensive evaluations of health-related policies should consider likely interactions with industry marketing strategies to fully understand potential impacts.

 Discussions on policies to tackle high prevalence of obesity and unhealthy diets, particularly among children have been ongoing in the United Kingdom for more than a decade. Taxation of sugary beverages became a centre point of these discussions once evidence of its intended impact on purchases started to emerge from early-adopting countries.^1^ In 2016 the UK government announced its plans for Sugary Drinks Industry Levy which came into effect in 2018.^2^ This created a two-year buffer period to encourage the industry to reformulate drinks to contain less sugar and thereby face the lower rate levy or avoid the levy altogether. The Soft Drink Industry Levy (SDIL) can now be called a success in that it has reduced the amount of sugar in sugary drinks. One of the first evaluation studies found a 34 percent point reduction in availability of soft drinks that had enough sugar (>5 g/100 mL) to be subject to the levy while no change was observed in the number of products or the size of available products.^3^ This is clearly a result of widespread reformulation of drinks. What is interesting in the UK context and why reformulation has been such a success is the relatively high consumer acceptability of artificially sweetened drinks which already pre-levy made up nearly half of the soft drink market.^4,5^

 Forde et al^6^ contribute to the literature analysing the effects of the SDIL with a qualitative study that seeks to understand industry marketing responses to the levy. They have interviewed 18 stakeholders from industry, academia and civil society with experience in strategic decision-making or marketing practices of soft drinks companies. Through the analysis of material from interviews they propose a theoretical framework of marketing decision-making in response to the SDIL and report common themes that emerge from the data. They find and discuss the heterogeneity of the possible industry responses ranging from reformulation to changes in prices, promotions, placement, product size, messaging, campaigns, introduction of new products or brands, or distribution. The extent to which any of this is done by different companies depends on what they suggest as ‘external’ (ie, competitors’ actions, consumer and policy trends, supplier and retailer relationships) and ‘internal’ (ie, size of the business or portfolio, strength and identity of the brand context, capacity and willingness, reputational concerns) context.

 Forde et al^6^ study, like a small number of recent papers,^7,8^ highlights the need for more nuanced conceptualizations of food marketing and corporate strategy within the public health literature. The framework that the authors draw is therefore a useful and welcome addition to this limited literature on understanding the likely industry responses to health-related food policies. The authors rightly note that there is potential to broaden the use of the framework beyond the sugary drinks levy by testing it on other health related food policies. In the following sections of this commentary our aim is to give some suggestions that may broaden the application of the framework for wider use.

 First, the applicability of the framework could broaden if the study’s themes were analysed more in-depth in relation with the existing literature on corporate marketing strategy. While the authors focus around the ‘four P’ (product, prices, promotion, and placement) or also known as the marketing mix, it would be useful to consider (and incorporate as applicable) other relevant concepts that may have not come up in interviews. These include concepts such as barriers to imitation, the idea of industry leaders and outsiders, consumers’ brand loyalty and product substitutability, asymmetry of information, and role of credence goods among others.^9-12^ This could help in improving our understanding of how the response to health-related food policies sits within the broader marketing strategies of the individual company or the industry as a whole. Second, the applicability of the framework could also be expanded by explicitly considering interactions among the different contextual factors. For example, it is the interaction between consumer behaviour, brand reputation, and competitor behaviour that determines whether the company chooses a plain price competition strategy, a differentiation strategy involving credence goods and going beyond the ‘problem of sugar,’ a re-branding or portfolio diversification or a large and highly publicised marketing investment, in order to deter imitation by potential competitors.^13^

 Crucially, as the authors rightly conclude, it is important to be able to avoid situations where marketing activities can effectively undermine the intended public health goals of policies. For example, if sugary drinks are taxed but a subsequent aggressive promotion campaign drives prices down then effects from the tax are likely to be diluted. What we do not know and what we do not learn from the article is to what extent any of the marketing strategies happened in the United Kingdom as a response to the SDIL. In fact, this is little researched area altogether. A recent paper from the United States, which to our knowledge is the first one to explicitly examine promotion activity following soda taxes, finds for example that in the United States promotion frequency and promotion depth decreased post soda-taxes by 28% and 11%, respectively.^14^ This suggests that the effects of marketing actions could in this case be supportive of the public health goals rather than negating their effects.Similar quantitative analyses of promotion activity as well as other types of marketing responses would be very informative in the United Kingdom and in other countries that have implemented a tax on sugary drinks (or unhealthy foods). Wide ranging evidence is important as the tax structures, consumer behaviour, market structures as well as other contextual factors and interactions between are very likely to be country specific.

 Given the lengthy ongoing debate around obesity prevention and policies targeting sugar drinks, it is perhaps surprising that relatively little is known about what drives industry marketing responses. Marketing actions around sugar and sugary drinks had already started before SDIL was announced but really took off from around this time^7^ and in combination with other policy interventions such as voluntary sugar reduction programme spilled over also to foods.^2^ Our own research^7^ has identified and characterised a series of proactive and defensive health-related marketing strategies used by the food industry. Health-oriented narrative and actions seem to help some companies keep one step ahead but for others to survive the external pressure from policy or competitors and simply stay alive. Some of the actions (strategies), as mentioned above, support policy (or more broadly public health) goals while others negate them and classifying these as such could be another useful angle for the framework to understand the effect of marketing actions. Policy supportive marketing actions tend to be more structural changes such as reformulation, clear labelling, or changes in portion size of best-selling products. Policy negating marketing actions are more commonly less structural and therefore require greater agency from the consumer while seemingly still making positive changes. These are for example introducing low-sugar alternatives alongside the main sugary products but not replacing them, creating smaller pack sizes but selling them in multi-packs or focusing on other product characteristics (eg, added vitamins, protein, natural or organic ingredients while still containing large amounts of sugar) leaning away from sugar or to differentiate from competitors. Pricing and promotion decisions however can fall into either category depending on what is seen as the best course of action.

 Finally, it is important to emphasise, as also mentioned by Forde et al^6^ in their discussion, the relative importance of different companies and thus the power to influence population health considering the consumption base. Big changes by small companies are important in that they can collectively be a driving force to make big companies change their strategies but on their own their impact on population level is minimal if market share is small. Thus, understanding the actions by large, market leading companies and brands is really important. Beyond using data that is available for research, engaging companies, particularly big ones, in academic work however is difficult. It is clearly illustrated in Forde et al^6^ study as they were able to interview only 9% of the people they had contacted from industry and only one of the six interviewed had experience in working in a large soft drink manufacturer. Some valuable evidence on internal company strategies can come from leaked documents^15,16^ but there is also scope for facilitated (eg, by relevant departments in the government or third sector) collaboration between industry and academia. Changes in regulation regarding data sharing are, of course, another option, but are probably best understood as a last resort strategy. This might only be feasible for specific types of data which have clear public health relevance and are hard to manipulate.

 The timing to expand our understanding of food industry marketing responses could not be more relevant. New health-related food policies banning placement of high fat, sugar and salt foods to end-of-aisles, checkouts and near store entrances in supermarkets came into effect in October 2022.^17^ Marketing efforts to adapt to the policy, which is likely to have bigger effect than SDIL had because it affects a much wider range of products, has been ongoing for a while. As the industry constantly monitors and adapts its environment in which it is operating so should health policy-makers. Measures of policy impact, such as changes in sales or even health impacts can miss a lot if the likely effects are actively counteracted, including prior to policies coming to effect. Therefore, going forward, comprehensive evaluations of health-related policies should also consider a broader understanding of industry marketing actions.

## Ethical issues

 Not applicable.

## Competing interests

 Authors declare that they have no competing interests.

## Authors’ contributions

 Both authors contributed equally to this work.

## Funding

 SC is funded by a grant from the Wellcome Trust (grant number 205200) under its ‘Our Planet, Our Health’ initiative. LC is funded by a UK Medical Research Council fellowship (MR/P021999/1).

## References

[1] Colchero MA, Popkin BM, Rivera JA, Ng SW (2016). Beverage purchases from stores in Mexico under the excise tax on sugar sweetened beverages: observational study. BMJ.

[2] HM Government. Childhood Obesity: A Plan for Action. 2016. https://www.gov.uk/government/publications/childhood-obesity-a-plan-for-action. Accessed August 18, 2022.

[3] Scarborough P, Adhikari V, Harrington RA (2020). Impact of the announcement and implementation of the UK Soft Drinks Industry Levy on sugar content, price, product size and number of available soft drinks in the UK, 2015-19: a controlled interrupted time series analysis. PLoS Med.

[4] BSDA. Annual Report. 2021. https://www.britishsoftdrinks.com/write/MediaUploads/BSDA_Annual_Report_2021_FINAL.pdf. Accessed August 18, 2022.

[5] BSDA. Annual Report. 2016. https://www.britishsoftdrinks.com/write/MediaUploads/Publications/BSDA_Annual_report_2016.pdf. Accessed August 18, 2022.

[6] Forde H, Penney TL, White M, Levy L, Greaves F, Adams J (2022). Understanding marketing responses to a tax on sugary drinks: a qualitative interview study in the United Kingdom, 2019. Int J Health Policy Manag.

[7] Cuevas S, Patel N, Thompson C (2021). Escaping the Red Queen: health as a corporate food marketing strategy. SSM Popul Health.

[8] Wood B, Williams O, Nagarajan V, Sacks G (2021). Market strategies used by processed food manufacturers to increase and consolidate their power: a systematic review and document analysis. Global Health.

[9] Dickson PR, Ginter JL (1987). Market segmentation, product differentiation, and marketing strategy. J Mark.

[10] Fisher RJ (1991). Durable differentiation strategies for services. J Serv Mark.

[11] Porter ME. The Competitive Strategy: Techniques for Analyzing Industries and Competitors. New York: Free Press; 1980.

[12] Veeman M (2002). Policy development for novel foods: issues and challenges for functional food. Canadian J Agric Econ.

[13] Piazzai M, Wijnberg NM (2019). Product proliferation, complexity, and deterrence to imitation in differentiated-product oligopolies. Strateg Manag J.

[14] Keller K, Guyt J, Grewal R. Soda Taxes and Marketing Conduct. https://papers.ssrn.com/sol3/papers.cfm?abstract_id=4127264. Posted June 15, 2022.

[15] Lauber K, Rippin H, Wickramasinghe K, Gilmore AB (2022). Corporate political activity in the context of sugar-sweetened beverage tax policy in the WHO European Region. Eur J Public Health.

[16] Goltzman MK, Kammerer P. INFORM: EU Public Policy Developments in February & March 2016. DC Leaks Coca Cola Emails. https://www.industrydocuments.ucsf.edu/docs/fncl0226. Accessed January 18, 2023. Published 2016.

[17] HM Government. Restricting Promotions of Products High in Fat, Sugar or Salt by Location and by Volume Price: Implementation Guidance. 2022. https://www.gov.uk/government/publications/restricting-promotions-of-products-high-in-fat-sugar-or-salt-by-location-and-by-volume-price/restricting-promotions-of-products-high-in-fat-sugar-or-salt-by-location-and-by-volume-price-implementation-guidance. Accessed August 18, 2022.

